# Temporal-spatial analysis of a foot-and-mouth disease model with spatial diffusion and vaccination

**DOI:** 10.3389/fvets.2022.952382

**Published:** 2022-12-05

**Authors:** Junyuan Yang, Xiaoyan Wang, Kelu Li

**Affiliations:** ^1^Complex Systems Research Center, Shanxi University, Taiyuan, China; ^2^Shanxi Key Laboratory of Mathematical Techniques and Big Data Analysis on Disease Control and Prevention, Shanxi University, Taiyuan, China; ^3^School of Information, Shanxi University of Finance and Economics, Taiyuan, China; ^4^School of Mathematics and Information Science, Henan Normal University, Xinxiang, China

**Keywords:** foot-and-mouth disease, the basic reproduction number, vaccination coverage, diffusion, latent period

## Abstract

Foot-and-mouth disease is an acute, highly infectious, and economically significant transboundary animal disease. Vaccination is an efficient and cost-effective measure to prevent the transmission of this disease. The primary way that foot-and-mouth disease spreads is through direct contact with infected animals, although it can also spread through contact with contaminated environments. This paper uses a diffuse foot-and-mouth disease model to account for the efficacy of vaccination in managing the disease. First, we transform an age-space structured foot-and-mouth disease into a diffusive epidemic model with nonlocal infection coupling the latent period and the latent diffusive rate. The basic reproduction number, which determines the outbreak of the disease, is then explicitly formulated. Finally, numerical simulations demonstrate that increasing vaccine efficacy has a remarkable effect than increasing vaccine coverage.

## 1. Introduction

The foot-and-mouth disease virus (FMDV), a spherical, capsule-free, single-stranded RNA virus, is an infectious disease that causes foot-and-mouth disease (FMD). Both domestic and wild animals with cloven hooves are susceptible to FMDV infection (1). FMD frequently causes dairy cattle to produce less milk and beef cattle and pigs to lose weight. The efficacy of vaccination against infection is frequently weakened by temporal and spatial variations in FMDV antigenicity (2). Consequently, the presence of FMD poses a significant barrier to international trade, has a negative impact on the livestock industry, and results in significant economic losses for animal products (3). Therefore, the World Organization for Animal Health (WOAH) has ranked FMD as the top animal disease.

Animals with a clinical infection always have FMDV in their excretions and secretions, contaminating the environment (4). There are three different types of FMD transmission routes: (1) Direct transmission: the infection spreads through direct contact between infected animals and naive animals (5); (2) Indirect transmission: the infection spreads through indirect contact *via* fomites (6); (3) Airborne transmission: the transmission of virus-carrying particles through aerosols (7). It has been shown that FMDV can survive various conditions and maintain a longer survival cycle. Therefore, it is important to acknowledge that contaminated environments can transmit FMD infection to animals as a risk factor. Colenutt et al. found that the $R_{0}$ estimated reproduction number is 1.65, which is significantly lower than the amount for direct animal to animal transmission. However, it would be sufficient to sustain an outbreak even if control measures to prevent direct transmissions, including animal movement and culling restrictions, are implemented (6).

Vaccination is a very effective measure of preventing FMD outbreaks in field conditions and lab settings (8). Evidence has shown that FMDV was radically eliminated in cattle after vaccination (4, 9). It can effectively lower the cost of agricultural production and the cost of health from an economic standpoint. According to reports, FMD has seven serotypes: A, O, C, Asial, SAT1, SAT2, and SAT3, all of which are highly mutagenic (2). Generally, vaccination with one serotype of these seven strains does not protect against other serotypes and does not provide complete protection from a single shot. Hence, to limit FMD infection, emergency ring vaccination and culling of infected animals have been executed. Vaccinating and restricting the movement of infected animals and their products is crucial when dealing with an outbreak of FMD transmission (10, 11). Several studies have quantified the efficacy of FMD vaccinations and evaluated the comprehensive economic consequences from a statistical point of view. The major concern is whether vaccinating all susceptible animals is required to limit the spread of FMD or if vaccinating only against certain agents could be adequate. It is essential to employ mathematical models to qualitatively assess the comprehensive efficacy of FMD vaccination and provide guidance for policymakers. For example, Mushayabasa et al. proposed a basic compartment model to investigate the effects of vaccination and the impact of seasonal conditions on the spread of foot-and-mouth disease (12). De Rueda et al. estimated that in mixed cattle-sheep populations with at least 14% of cattle, vaccination of cattle is sufficient to lower $R_{0}$ to be less than 1 (8). The causes of FMD outbreaks have been explained in detail by Lyons et al. to demonstrate the effectiveness of vaccines for FMD control (13).

Many dynamic models have been explored for examining long-term FMD behaviors according to their transmission mechanisms. Mathematical models can be used to build preparedness plans in advance of an outbreak epidemic, anticipate outbreaks, and evaluate the efficacy of control measures. Researchers proposed several models to forecast FMD development trends in response to the UK's 2001 FMD epidemics (14–17). For instance, Ferguson et al. built an empirical model to forecast changes in the foot-and-mouth disease outbreak (18). Keeling et al. used the Cambridge–Edinburgh model to address the long tail property of foot-and-mouth disease cases in the UK in 2001 (19, 20). Morris et al. developed the inter-spread model to evaluate the transmission of temporal-spatial foot-and-mouth disease (21). Lewis and Ward adopted a logistic regression model to ascertain whether a collection of explanatory factors was associated with an outbreak of foot-and-mouth disease (22). Ringa and Bau created a pair approximation model to examine the role of vaccination in the optimal long-term prevention of the spread of foot-and-mouth disease (23). Most of these models ignore animal heterogeneities and assume all animals are mixed homogeneously. Jolles et al. found that FMD viruses cannot persist among infected hosts without environmental transmission through experimental and theoretical methods (24). Colenutt et al. found that environmental transmission has been linked to long-lasting FMD outbreaks (6). Bravo de Rueda et al. quantified the FMDV transmission process and showed that the environment is responsible for approximately half of FMDV transmission (25).

Animal movements significantly impact the FMD transmission pattern since it was revealed that FMD had displayed geographical diversity. Mathematical models must be used to reveal the mechanisms of spatial transmission for FMD infection. Three basic models are being used to analyze such temporal-spatial features. Spatial diffusive models investigate the temporal-spatial dynamics described by partial differential equations (26). The main focus of percolation theory is the impact on the farming landscape. Network models examine short- or long-distance transmissions starting from stochastic events (27). The information for the last two models was frequently obtained from a statistical physics point of view (28).

In this paper, we build a linked model of FMD transmission from animal to animal and from FMD virus to animal with an age-space structure. We offer a diffusive mathematical model with partial immunity from vaccination, which implies that the vaccinated animals may catch infection again once they come in contact with the infected ones. The FMD vaccine cannot provide total immunity against FMD transmission. According to numerical analysis, increasing vaccine efficacy has a greater impact than increasing vaccination coverage.

## 2. Method

The qualitative analysis of the evolution of FMDV transmission relies heavily on mathematical models since they offer a conceptual framework for understanding a particular system's language and making a large-scale prediction. FMDV prevalence is significantly influenced by spatial effects, animal movements, and vaccine efficacy. Identifying the FMDV transmission mechanisms in the UK can be done with the help of a spatial diffusion model (19, 20). In this paper, we used a spatial diffusion model to investigate the efficacy of the vaccination against FMDV infection. The model complies with the “compartmental concepts” proposed by Kermack and McKendrick (29), which couples with the Laplace operator Δ to describe an animal's random movements. This model provides the most accurate representation of spatial FMDV propagation due to diffusion.

### 2.1. Model formulation

The main concern of this paper is to reveal the temporal and spatial patterns of FMD transmission. According to the compartmental modeling rules, we categorize the total cattle population *N*(*t, x*) into three subgroups: susceptible animals, vaccinated animals, and infected animals. *S*(*t, x*)(*V*(*t, x*)) denotes the space density of susceptible (vaccinated) animals at time *t* in position $x\in \bar{\text{Ω}},$ where Ω⊂ℝ^*n*^ is a bounded subset of ℝ^*n*^.

The early detection of the incursion as well as the ability to efficiently trace and identify animals that have been exposed to the source of infection is crucial for curbing FMD transmission. There exists a high-risk period from the first infection to the detected first case, which lasts about 0.5 days after susceptible animals contact infected animals. During such period, there are potentially subtle or unapparent clinical signs of infection and it causes an underestimation of the infection. For the description of such period, we employ an age of infection to investigate the preclinical transmission process. *i*(*t, a, x*) represents the age-space density of infected animals with since infection age *a*, at time *t* in position *x*. *B*(*t, x*) denotes the space density of foot-and-mouth virus (FMDV) in a contaminant environment at time *t* in position *x*∈Ω.

We hypothesize that susceptible cattle directly contact infected cattle and get an infection at rate β(*a*), where *a* is the age since infection, and moreover, susceptible cattle can get infection indirectly contacting by fomites in the contaminated environment at rate $\frac{\beta_{B}}{\kappa +B\left(t,x\right)}$. Conversely, we assume that vaccinated cattle can be infected both by infected cattle and FMDV at a discount rate σ compared with the original infection. The infection force is defined by


$$\lambda \left(t,x\right)=\left(\overset{\infty }{\underset{0}{\int }}\beta \left(a\right)i\left(t,a,x\right)da+\frac{\beta_{B}B\left(t,x\right)}{\kappa +B\left(t,x\right)}\right).$$

**Figure 1:**
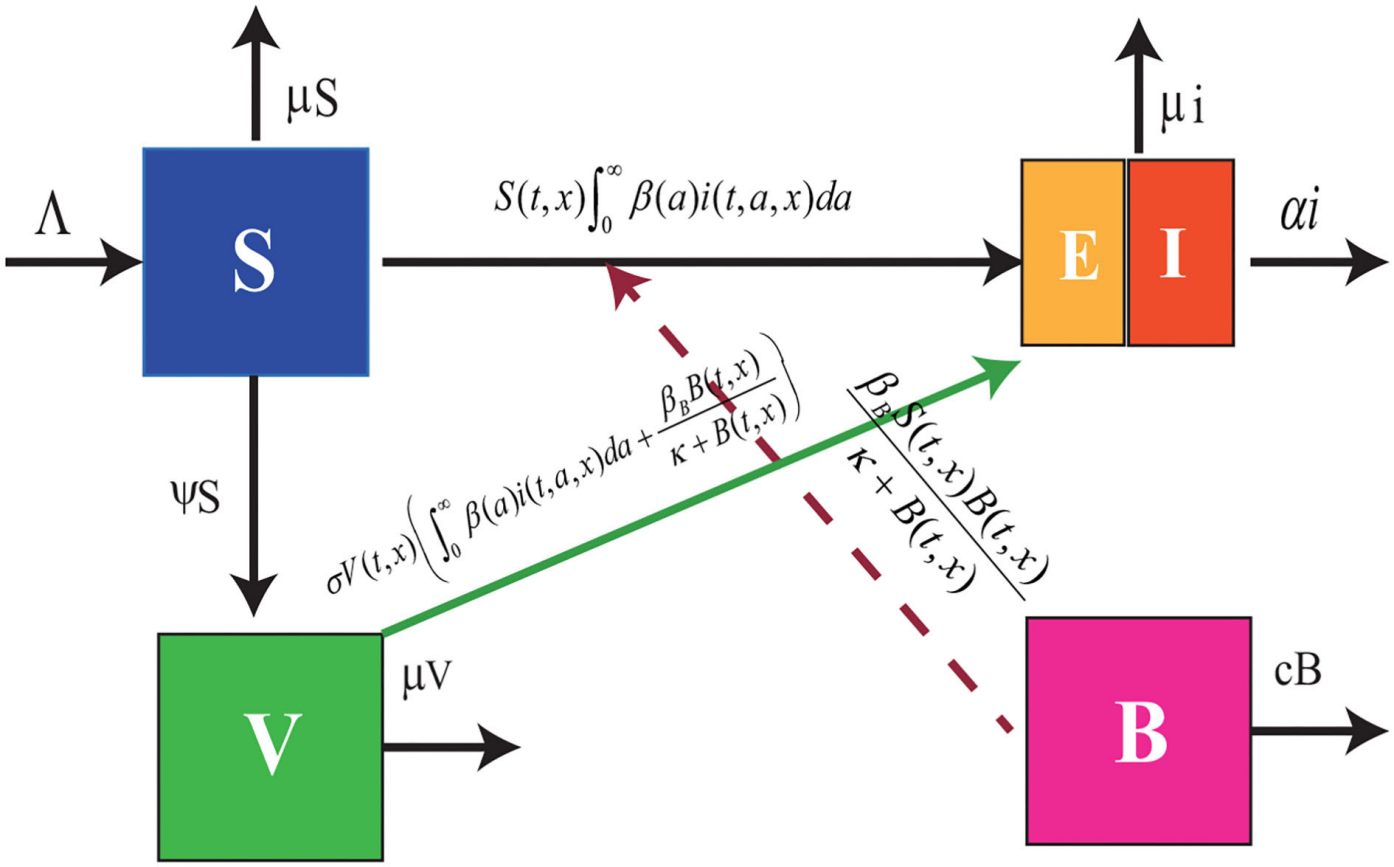
Flowchart of model (Equation 1). The blue box denotes the susceptible cattle, the green box represents the vaccinated cattle, the purple box stands for the density of FMDV in the contaminated environment, and the combined box denotes the infected cattle including the exposed cattle and the symptomatic cattle.

Motivated by the above, the mechanisms of a foot-and-mouth disease model are characterized in the following equations (see Figure 1):


(1) $$\{\begin{array}{l} \frac{\partial S(t,x)}{\partial t}=d_{S}\text{Δ}S(t,x)+\text{Λ}-(\mu +\psi )S(t,x)\\ -S(t,x)\lambda (t,x),x\in \text{Ω},\\ \frac{\partial V(t,x)}{\partial t}=d_{V}\text{Δ}V(t,x)+\psi S(t,x)-\mu V(t,x)\\ -\sigma V(t,x)\lambda (t,x),x\in \text{Ω},\\ \frac{\partial i(t,a,x)}{\partial t}+\frac{\partial i(t,a,x)}{\partial a}=d_{i}(a)\text{Δ}i(t,a,x)\\ -(\mu +\alpha (a))i(t,a,x),x\in \text{Ω},\\ i(t,0,x)=(S(t,x)+\sigma V(t,x)\lambda (t,x),x\in \text{Ω},\\ \frac{\partial B(t,x)}{\partial t}=d_{B}\text{Δ}B(t,x)+\int_{0}^{\infty }p(a)i(t,a,x)da\\ -cB(t,x),x\in \text{Ω},\\ \frac{\partial S(t,x)}{\partial n}=\frac{\partial V(t,x)}{\partial n}=\frac{\partial i(t,a,x)}{\partial n}=\frac{\partial B(t,x)}{\partial n}=0,x\in \partial \text{Ω}, \end{array}$$

where *d*_*j*_(*J* = *S, V, i, B*) denotes the diffusion coefficients of susceptible, vaccinated, infected animals, and foot-mouth viruses, Λ denotes the produce rate, μ and *c* denote slaughter rate of cattle and the degradation rate of FMDV, respectively. α(·) stands for the death rate caused by FMD. The infected animals release the FMDV into the environment at rate *p*(·). ∂/∂*n* denotes the derivative along the outward unite normal vector *n*.

By Theorem 1.5 in Pazy (33), the operator *d*_*j*_Δ(*j* = *S, V, i, B*) with the zero flux boundary condition generate the following compact and strongly positive semigroups


$$(T_{j}(t)[\phi ])(x)=\int_{\text{Ω}}{\text{Γ}}_{j}(t,x,y)\phi (y)dy,j=S,V,i,B.$$

where Γ_*j*_(*j* = *S, V, i, B*) are Green functions. Assume that the latent period is τ, then we can separate the infected cattle into two subgroups:


$$E(t,x)=\int_{0}^{\tau }i(t,a,x)da,\;I(t,x)=\int_{\tau }^{\infty }i(t,a,x)da,$$

where *E*(*t, x*) represents the space density of latent cattle at time *t* in position *x*, *I*(*t, x*) denotes the space density of infected cattle at time *t* and position *x*. Integrating the third equation of model (1) with its initial and boundary conditions, we have that


(2) $$i(t,a,x)=\left\{ \begin{array}{l} \int_{\text{Ω}}{\text{Γ}}_{i}(a,x,y)i(t-a,0,y)dy\pi (a),\;t\geq a,\\ \int_{\text{Ω}}{\text{Γ}}_{i}(a,x,y)i_{0}(a-t,y)dy\frac{\pi (a)}{\pi (a-t)},\;t<a, \end{array}\right.$$

where


$$\pi (a)=e^{-\int_{0}^{a}(\mu +\alpha (s))ds}$$

represents the probability of an infected animal survives until infect age *a*. If we set


$$\begin{array}{l} d_{i}(a)=\{\begin{array}{l} d_{E},\;0\leq a\leq \tau ,\\ \;d_{I},\;\tau <a<\infty , \end{array}\;\beta (a)=\{\begin{array}{l} \beta_{E},\;0\leq a\leq \tau ,\\ \beta ,\;\tau <a<\infty , \end{array}\\ \alpha (a)=\{\begin{array}{l} \alpha_{E},\;0\leq a\leq \tau ,\\ \alpha ,\;\tau <a<\infty , \end{array}\;p(a)=\{\begin{array}{l} p_{E},\;0\leq a\leq \tau ,\\ \;p,\;\tau <a<\infty . \end{array} \end{array}$$

Based on the above assumptions on β(·), *d*_*i*_(·), and α(·), then the evolution of the latent and infected cattle satisfies


(3) $$\begin{array}{l} \frac{\partial E(t,x)}{\partial t}=\;d_{E}\text{Δ}E(t,x)-(\mu +\alpha_{E})E(t,x)\\ \;-\int_{\text{Ω}}{\text{Γ}}_{i}(\tau ,x,y)(S(t-\tau ,x)+\sigma V(t-\tau ,x))\\ \;\times (\beta_{E}E(t-\tau ,y)+\beta_{I}I(t-\tau ,y)\\ \;+\frac{\beta_{B}B(t-\tau ,x)}{1+\alpha B(t-\tau ,y)})dye^{-(\mu +\alpha_{E})\tau }\\ \;+(S(t,x)+\sigma V(t,x))\\ \;\left(\beta_{E}E(t,x)+\beta I(t,x)+\frac{\beta_{B}B(t,x)}{1+\alpha B(t,x)}\right),\\ \frac{\partial I(t,x)}{\partial t}=\;d_{I}\text{Δ}I(t,x)da-(\mu +\alpha )I(t,x)\\ \;+\int_{\text{Ω}}{\text{Γ}}_{i}(\tau ,x,y)(S(t-\tau ,x)+\sigma V(t-\tau ,x))\\ \;\times \;(\beta_{E}E(t-\tau ,y)+\beta_{I}I(t-\tau ,y)\\ \;+\frac{\beta_{B}B(t-\tau ,x)}{1+\alpha B(t-\tau ,y)})dye^{-(\mu +\alpha )\tau }. \end{array}$$

The detailed derivations of E and I are enclosed in Appendix A. From Equation (3), it is easy to see that the compartment *E* is decoupled, but the latent information is inclosed in the term


$$\begin{array}{l} \;\int_{\text{Ω}}{\text{Γ}}_{i}(\tau ,x,y)(S(t-\tau ,x)+\sigma V(t-\tau ,x))\\ \left(\beta_{I}I(t-\tau ,y)+\frac{\beta_{B}B(t-\tau ,x)}{1+\alpha B(t-\tau ,y)}\right)dye^{-(\mu +\alpha_{E})\tau }, \end{array}$$

where we have assumed that the latent cattle has no infected ability. Replacing *i* in Equation (1) and ignoring equation *E*, one arrives at


(4) $$\left\{ \begin{array}{l} \frac{\partial S(t,x)}{\partial t}=d_{S}\text{Δ}S(t,x)+\text{Λ}-(\mu +\psi )S(t,x)\\ -S(t,x)\left(\beta I(t,x)+\frac{\beta_{B}B(t,x)}{\kappa +v(t,x)}\right),x\in \text{Ω},\\ \frac{\partial V(t,x)}{\partial t}=d_{V}\text{Δ}V(t,x)+\psi S(t,x)-\mu V(t,x)\\ -\sigma V(t,x)\left(\beta I(t,x)+\frac{\beta_{B}V(t,x)}{\kappa +B(t,x)}\right),x\in \text{Ω},\\ \frac{\partial I(t,x)}{\partial t}=d_{I}\text{Δ}I(t,x)da-(\mu +\alpha )I(t,x)\\ +\int_{\text{Ω}}{\text{Γ}}_{i}(\tau ,x,y)(S(t-\tau ,x)+\sigma V(t-\tau ,x))\;\\ \;\times \left(\beta_{I}I(t-\tau ,y)+\frac{\beta_{B}B(t-\tau ,x)}{1+\alpha B(t-\tau ,y)}\right)dye^{-(\mu +\alpha )\tau },\\ x\in \text{Ω},\\ \frac{\partial B(t,x)}{\partial t}=d_{B}\text{Δ}B(t,x)+pI(t,x)-cB(t,x),x\in \text{Ω},\\ \frac{\partial S(t,x)}{\partial n}=\frac{\partial V(t,x)}{\partial n}=\frac{\partial i(t,a,x)}{\partial n}=\frac{\partial B(t,x)}{\partial n}=0,x\in \partial \text{Ω}, \end{array}\right.$$

where we have assumed that *p*_*E*_ = 0 suggesting that latent cattle do not release the FMDV into the environment. In what follows, we will focus on the efficacy of vaccination and the diffusion of the latent cattle on the temporal-spatial patterns of FMD transmission.

### 2.2. Basic reproduction number

The basic reproduction is the average number of secondary cases produced by an infected individual at a completely susceptible environment during his infectious period, which provides an overall measure of the potential for transmission of an infection in a population. Generally, if it is less than one, the disease dies out; otherwise, it invades the host population.

Lemma 0.3 in Appendix B implies that system (Equation (4)) has a disease-free steady state $E_{0}=\left(S^{0},V^{0},0,0\right)=\left(\frac{\text{Λ}}{\mu +\psi },\frac{\psi \text{Λ}}{\mu \left(\mu +\psi \right)},0,0\right)$. Linearizing system (Equation (4)) around the disease-free equilibrium *E*_0_, we obtain


(5) $$\left\{ \begin{array}{l} \frac{\partial I(t,x)}{\partial t}=d_{i}\text{Δ}I(t,x)-(\mu +\alpha )I(t,x)\\ +(S^{0}+\sigma V^{0})\int_{\text{Ω}}{\text{Γ}}_{i}(\tau ,x,y)\;\\ \;\times \left(\beta_{I}I(t-\tau ,y)+\frac{\beta_{B}B(t-\tau ,y)}{\kappa }\right)dye^{-(\mu +\alpha )\tau },x\in \text{Ω},\\ \frac{\partial B(t,x)}{\partial t}=d_{B}\text{Δ}B(t,x)+pI(t,x)-cB(t,x),x\in \text{Ω},\\ \frac{\partial I(t,x)}{\partial n}=\frac{\partial B(t,x)}{\partial n}=0,x\in \partial \text{Ω}. \end{array}\right.$$

Let us introduce a newly infection operator


$$F\left[\phi \right]\left(x\right)=\left(F_{1}\left[\phi \right],F_{2}\left[\phi \right]\right)\left(x\right),\;\forall \phi =\left(\phi_{3},\phi_{4}\right)\in Y^{2},x\in \bar{\text{Ω}},$$

where


$$\begin{array}{l} F_{1}[\phi ](x)=(S^{0}+\sigma V^{0})\int_{\text{Ω}}{\text{Γ}}_{i}(\tau ,x,y)\left(\beta_{I}\phi_{3}(y)+\frac{\beta_{B}}{\kappa }\phi_{4}(y)\right)\\ \;dye^{-(\mu +\alpha )\tau },F_{2}=p\phi_{3}(x). \end{array}$$

Moreover, let us introduce a transition operator


$$B\left[\phi \right]\left(x\right)=\left(B_{1}\left[\phi \right],B_{2}\left[\phi \right]\right)\left(x\right),\;\forall \phi \in Y^{2},$$

where


$$B_{1}\left[\phi \right]=d_{i}\text{Δ}\phi_{3}\left(x\right)-\left(\mu +\alpha \right)\phi_{3}\left(x\right),B_{2}\left[\phi \right]=d_{B}\text{Δ}\phi_{4}\left(x\right)-c\phi_{4}\left(x\right).$$

Besides, the transit operator *B* generates the following positive and compact semigroup


$$\begin{array}{l} T([\phi ](x))(t)=(e^{-(\mu +\alpha )t}\int_{\text{Ω}}{\text{Γ}}_{i}(t,x,y)\phi_{3}(y)dy,e^{-ct}\\ \;\int_{\text{Ω}}{\text{Γ}}_{B}(t,x,y)\phi_{4}(y)dy{)}^{T},\forall t\in {\mathbb{R}}_{+}. \end{array}$$

Then


$${\left(-B\right)}^{-1}[\phi ](x)=\int_{\infty }^{0}T[\phi ](t)dt.$$

Hence, the next-generation operator *G* can be defined by


$$\begin{array}{l} G[\phi ](x)=F{\left(-B\right)}^{-1}[\phi ](x)=(F_{1}{\left(-B_{1}\right)}^{-1}[\phi ](x),\\ \;F_{2}{\left(-B_{2}\right)}^{-1}[\phi ](x){)}^{T}, \end{array}$$

where


$$\begin{array}{l} F_{1}{\left(-B_{1}\right)}^{-1}[\phi ](x)=(S^{0}+\sigma V^{0})\int_{0}^{\infty }\int_{\text{Ω}}{\text{Γ}}_{i}(\tau ,x,y)(\beta_{I}e^{-(\mu +\alpha )t}\\ \;\int_{\text{Ω}}{\text{Γ}}_{i}(t,x,y)\phi_{3}(y)dy\\ \;+\frac{\beta_{B}}{\kappa }e^{-ct}\int_{\text{Ω}}{\text{Γ}}_{B}(t,x,y)\phi_{4}(y)dy)\\ \;dydte^{-(\mu +\alpha )\tau },\\ F_{2}{\left(-B_{2}\right)}^{-1}[\phi ](x)=p\int_{0}^{\infty }e^{-(\mu +\alpha )t}\int_{\text{Ω}}{\text{Γ}}_{i}(t,x,y)\phi_{4}(y)dydt. \end{array}$$

Therefore, the basic reproduction number is defined by


$$R_{0}=\rho \left(G\right)$$

From the property of Γ_*j*_, we have concluded that the next operator *G* is positive and compact. Employing Krein–Rutman Theorem, $R_{0}$ is a positive eigenvalue with respect to a positive eigenvector ϕ, which suggests that


$$G\left[\phi \right]\left(x\right)=R_{0}\left[\phi \right]\left(x\right).$$

Letting ϕ = 1, then


(6) $${ℛ}_{0}=\frac{(S^{0}+\sigma V^{0})\frac{\beta }{\mu +\alpha }e^{-(\mu +\alpha )\tau }+\sqrt{{\left(S^{0}+\sigma V^{0}\right)}^{2}\frac{\beta^{2}}{{\left(\mu +\alpha \right)}^{2}}e^{-2(\mu +\alpha )\tau }+4(S^{0}+\sigma V^{0})\frac{\beta_{B}}{c\kappa }\frac{p}{\mu +\alpha }e^{-(\mu +\alpha )\tau }}}{2}.$$

From the epidemiological view of points, we introduce the other reproduction number by


(7) $$\begin{array}{l} {\hat{ℛ}}_{0}=(S^{0}+\sigma V^{0})\left(\frac{\beta }{\mu +\alpha }+\frac{\beta_{B}p}{\kappa (\mu +\alpha )c}\right)e^{-(\mu +\alpha )\tau }\\ \;={\hat{ℛ}}_{0a}+{\hat{ℛ}}_{0b}, \end{array}$$

where


$$\begin{array}{ll} {\hat{R}}_{0a}= & \left(S^{0}+\sigma V^{0}\right)\frac{\beta }{\mu +\alpha }e^{-\left(\mu +\alpha \right)\tau }, \end{array}$$

$$\begin{array}{ll} {\hat{R}}_{0b}= & \left(S^{0}+\sigma V^{0}\right)\frac{\beta_{B}p}{\kappa \left(\mu +\alpha \right)c}e^{-\left(\mu +\alpha \right)\tau } \end{array}$$

** Theorem 0.0.1**. Let $R_{0}$ and ${\hat{R}}_{0}$ be defined by Equations (6) and (7). The following statements are true:

(1) $R_{0}>1\Leftrightarrow {\hat{R}}_{0}>1;$
(2) $R_{0}<1\Leftrightarrow {\hat{R}}_{0}<1;$
(3) $R_{0}=1\Leftrightarrow {\hat{R}}_{0}=1.$

From what has been discussed, we return to give a detailed explanation for ${\hat{R}}_{0}.$ In fact, β*e*^−(μ+α)τ^ gives the average number of the secondary cases produced by one infected animal and it is still alive after the latent period τ. Hence, ${\hat{R}}_{0a}$ gives the average number of the secondary infected animals produced by an infected animal during its infectious period. Similarly, ${\hat{R}}_{0v}$ means that the average number of the secondary cases produced by a typical FMDV during its period.

## 3. Results

### 3.1. Theoretical results

In this section, we will show the basic reproduction number is a threshold index for disease extinction or persistence.

** Lemma 0.0.2**. For any $\phi \in C_{\tau }^{+},$ the following items hold.

(1) For any *t*∈ℝ_+_, *S*(*t*, ·)>0 and *V*(*t*, ·)>0. Moreover, there exists a positive value $\bar{\epsilon }$ such that

$$\lim\underset{t\to \infty }{\inf}S\left(t,\cdot \right)\geq \bar{\epsilon },\;\lim\underset{t\to \infty }{\inf}V\left(t,\cdot \right)\geq \bar{\epsilon },$$

(2) If there exists some *t*_0_≥0 such that *I*(*t*_0_, ·)≢0 or *B*(*t*_0_, ·)≢0, then

I(t,·)>0, B(t,·)>0,

*Proof*. In the proof of Lemma 0.1 in Appendix B, there exist two positive constants *T* and *M* such that for any $\left(t,x\right)\in \left(T,\infty \right)\times \bar{\text{Ω}}$


I(t,·)≤M,B(t,·)≤M,∀t>T.

In view of the first equation of (4), we note that


$$\begin{array}{l} \frac{\partial S(t,x)}{\partial t}\geq d_{S}\text{Δ}S(t,x)+\text{Λ}-(\mu +\psi \\ +(\beta +\beta_{B})M)S(t,x),(t,x)\in (T,\infty )\times \text{Ω},\\ \frac{\partial V(t,x)}{\partial t}\geq d_{V}\text{Δ}V(t,x)+\psi S(t,x)\\ -(\mu +\sigma (\beta +\beta_{B})M)V(t,x),(t,x)\in (T,\infty )\times \text{Ω},\\ \frac{\partial S(t,x)}{\partial n}=\frac{\partial V(t,x)}{\partial n}=0,x\in \partial \text{Ω}. \end{array}$$

By Lemma 4.1, the following system


$$\begin{array}{l} \frac{a\partial \bar{S}(t,x)}{\partial t}=d_{S}\text{Δ}\bar{S}(t,x)+\text{Λ}-(\mu +\psi \\ +(\beta +\beta_{B})M)\bar{S}(t,x),x\in \text{Ω},t\geq T_{B},\\ \frac{\partial \bar{V}(t,x)}{\partial t}=d_{V}\text{Δ}\bar{V}(t,x)+\psi \bar{S}(t,x)\\ -(\mu +\sigma (\beta +\beta_{B})M)\bar{V}(t,x),\\ \frac{\partial \bar{S}(t,x)}{\partial n}=\frac{\partial \bar{V}(t,x)}{\partial n}=0,x\in \partial \text{Ω}. \end{array}$$

has a unique equilibrium ${Ē}^{*}=\left({\bar{S}}^{0},{\bar{V}}^{0}\right)=\left(\frac{\text{Λ}}{\mu +\psi +\left(\beta +\beta_{B}\right)M},\frac{p\text{Λ}}{\left(\mu +\psi +\left(\beta +\beta_{B}\right)M\right)\left(\mu +\sigma \left(\beta +\beta_{B}\right)M\right)}\right)$ which is globally asymptotically stable in $C\left(\bar{\text{Ω}},\mathbb{R}\right)\times C\left(\bar{\text{Ω}},\mathbb{R}\right).$ By the standard parabolic comparison theorem, we conclude that


$$\lim\underset{t\to \infty }{\inf}S\left(t,\cdot \right)\geq {\bar{S}}^{0},\;\lim\underset{t\to \infty }{\inf}V\left(t,\cdot \right)\geq {\bar{V}}^{0}.$$

From Lemma 0.3 in the Appendix B, it follows that


(8) $$\left\{ \begin{array}{l} \frac{\partial I(t,x)}{\partial t}\leq d_{I}\text{Δ}I(t,x)-(\mu +\alpha )I(t,x),\\ \frac{\partial B(t,x)}{\partial t}\leq d_{B}\text{Δ}B(t,x)+pI(t,x)-cB(t,x). \end{array}\right.$$

The part (2) is a direct result of Theorem 3 and Theorem 4 in Protter and Weinberger (34) replacing *t* = 0 by *t* = *t*_0_.

** Theorem 0.0.3**. Suppose $R_{0}$ is defined in Equation (6). Then the following results hold.

(1) If $R_{0}<1,$ then the virus-free equilibrium *E*_0_ is globally asymptotically stable;
(2) If $R_{0}>1,$ then there exists a positive value ϵ>0 such that for all ϕ_3_(*x*)≢0 and ϕ_4_(*x*)≢0

$$\underset{t\to +\infty }{\lim\;\inf}I(t,x)\geq \epsilon ,\;\lim\inf V(t,x)\geq \epsilon$$

uniformly for all $x\in \bar{\text{Ω}}.$ Moreover, system (Equation 4) has at least one endemic equilibrium E^*^.

The detailed proof of Theorem 0.0.3 is enclosed in Appendix C.

### 3.2. Numerical results

In this section, we have conducted numerical examples to show some significant results. First, we fix some parameters in Table 1. Henec, we pick up


$${\text{Γ}}_{i}\left(\tau ,x,y\right)=\frac{2}{\pi }\overset{\infty }{\underset{n=1}{\sum }}\exp\left(-\left(n^{2}D_{L}+d+\alpha \right)\tau \right)\cos\left(nx\right)\cos\left(ny\right).$$

**Table 1:** List of parameter values.

**Parameters**	**Biological meanings**	**Values**	**Unit**	**References**
Λ	Produce rate	38,340	day^−1^	(30)
β_*B*_	The transmission rate from FMDV to cattle	1.3348 × 10^−6^	day^−1^	(30)
κ	The half-saturation concentration of the FMDV	10^8^	copies/cattle	(30)
μ	The slaughter rate	0.0018	day^−1^	(5)
α	The death rate due to FMD	1/3.5	day^−1^	(30)
*c*	The natural decay rate of FMDV	1/30	day^−1^	(31)
*p*	The pathogen production rate of an infected cattle	10^4.3^	day^−1^	(30)
ψ	the vaccinated rate	0.8	day^−1^	(32)
1−σ	the efficacy of vaccination	0.5	day^−1^	(11)
*d*_*S*_	The diffusion coefficient of susceptible cattle	0.0005	-	Assumed
*d*_*V*_	The diffusion coefficient of vaccinated cattle	0.0005	-	Assumed
*d*_*I*_	The diffusion coefficient of infected cattle	0.0003	-	Assumed
*d*_*B*_	The diffusion coefficient of FMDV	0.001	-	Assumed

The initial values are chosen as follows:


$$\begin{array}{c} \phi_{S}\left(\tau ,x\right)=4+\sin\left(x\right)\cos\left(\tau \right),\;\phi_{V}\left(\tau ,x\right)=4+\sin\left(x\right)\cos\left(\tau \right),\\ \phi_{I}\left(\tau ,x\right)=2+\sin\left(x\right)\cos\left(\tau \right),\;\phi_{B}\left(\tau ,x\right)=2+\sin\left(x\right)\cos\left(\tau \right). \end{array}$$

### 3.3. The dynamics of the system

**Figure 2:**
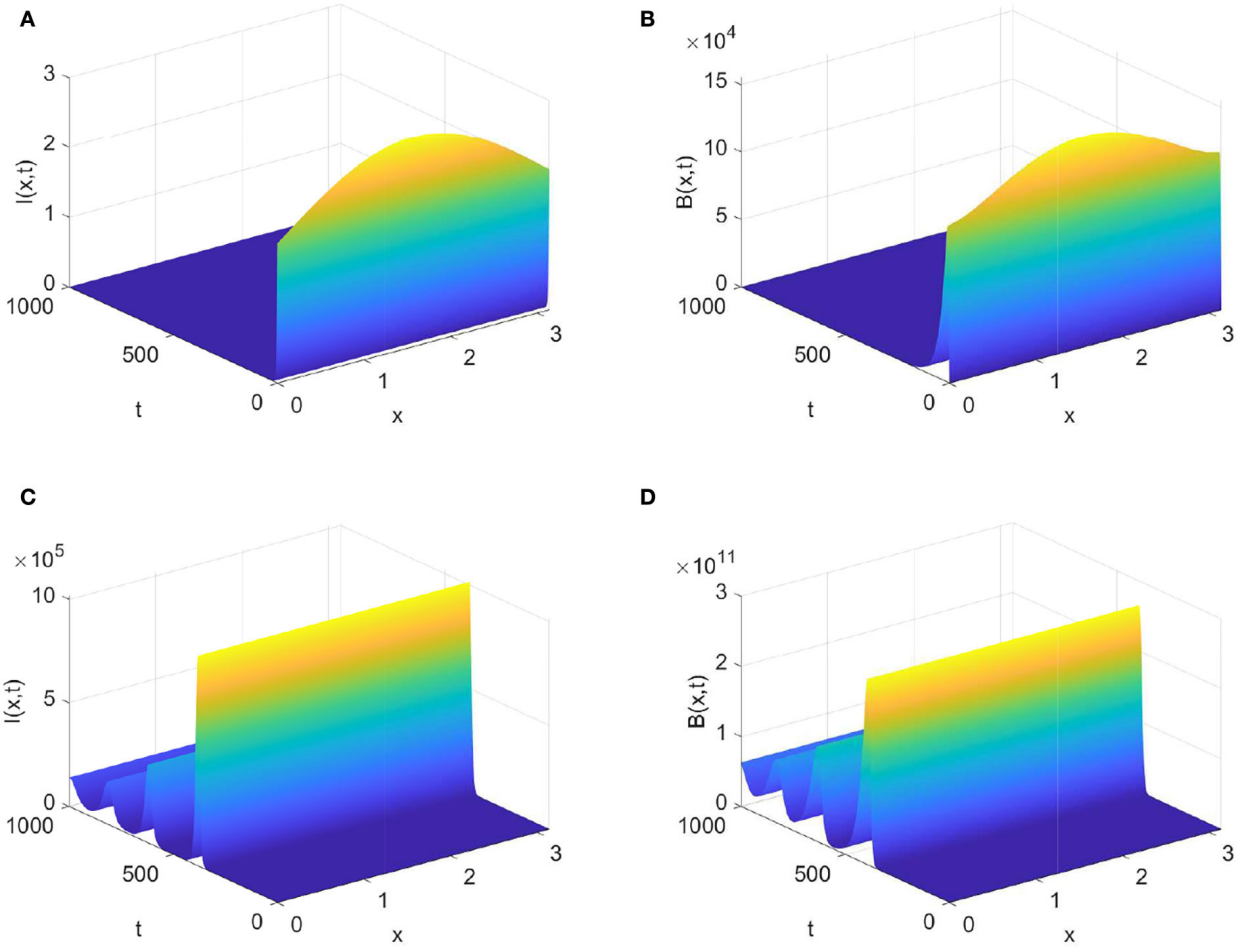
Evolution of infected animals and FMD virus with parameters in Table 1. **(A,B)** with $R_{0}\approx 0.8381<1.$ **(C,D)** with $R_{0}\approx 4.1778>1$.

Next, if we choose β = 3.0 × 10^−8^, then $R_{0}=0.8381<1.$ From Theorem 0.0.3 (1), it follows that the virus-free steady state *E*_0_ is globally attractive. Figures 2A,B show that the densities of infected animals and the FMD virus decay to zero as time goes to infinity. Enlarging β = 1.0 × 10^7^, we calculate $R_{0}=4.1778>1.$ Theorem 0.0.3 (2) ensures that the disease persists when $R_{0}>1$ and ϕ∈*W*_0_. Figures 2C,D display that the densities of *I*(*t, x*) and *B*(*t, x*) gradually decay to a positive distribution when time evolves.

### 3.4. Sensitivity analysis

**Figure 3:**
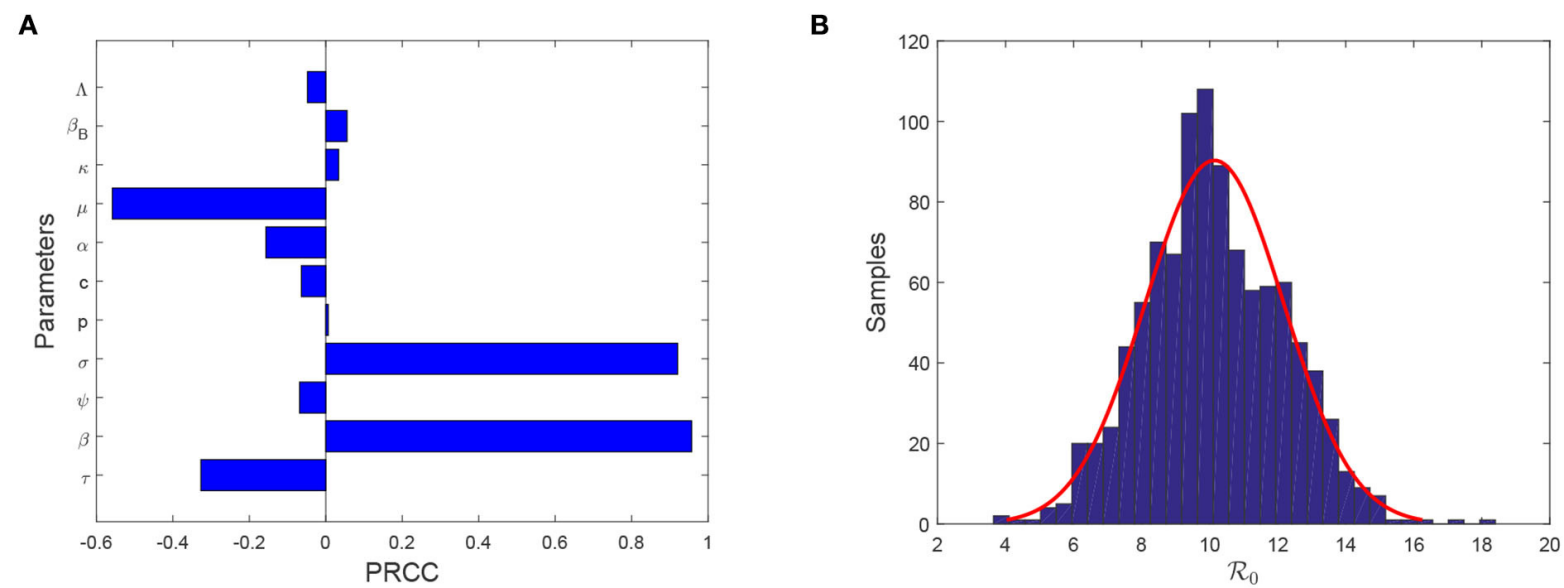
**(A)** Tornado plot of PRCC for model parameters associated with $R_{0}.$ **(B)** Samples of $R_{0}$.

Note that model (Equation 4) contains fifteen parameters. It is necessary to find which parameters are more sensitive than other parameters in affecting evolution of FMD infection. Theorem 0.0.3 shows that $R_{0}$ plays a significant role in determining the outbreak of FMD. Hence, we need to seek the sensitivity analysis of $R_{0}$ on each parameter. To achieve this aim, we select Latin hypercube sampling (LHS) to identify the rank of key factors that affect the basic reproduction number. In this process, we use partial rank correlation coefficient (PRCC) with 1,000 samples to give a tornado plot, which provides a visible figure to show the importance of every parameter's uncertainty. Figure 3A shows that reducing the transmission from animal to animal, improving the efficacy of vaccination, enlarging the curing rate, and lengthening the latent period are helpful for reducing the size of $R_{0}.$ Moreover, reducing transmission rate from animal to animal has the most importance than other control measures. The samples of $R_{0}$ converge a normal distribution with an average value 10.1404 [95% CI (10.014–1.02668)] and a variance 2.0369 [95% CI(1.9514–2.1304)] (see Figure 3B).

**Figure 4:**
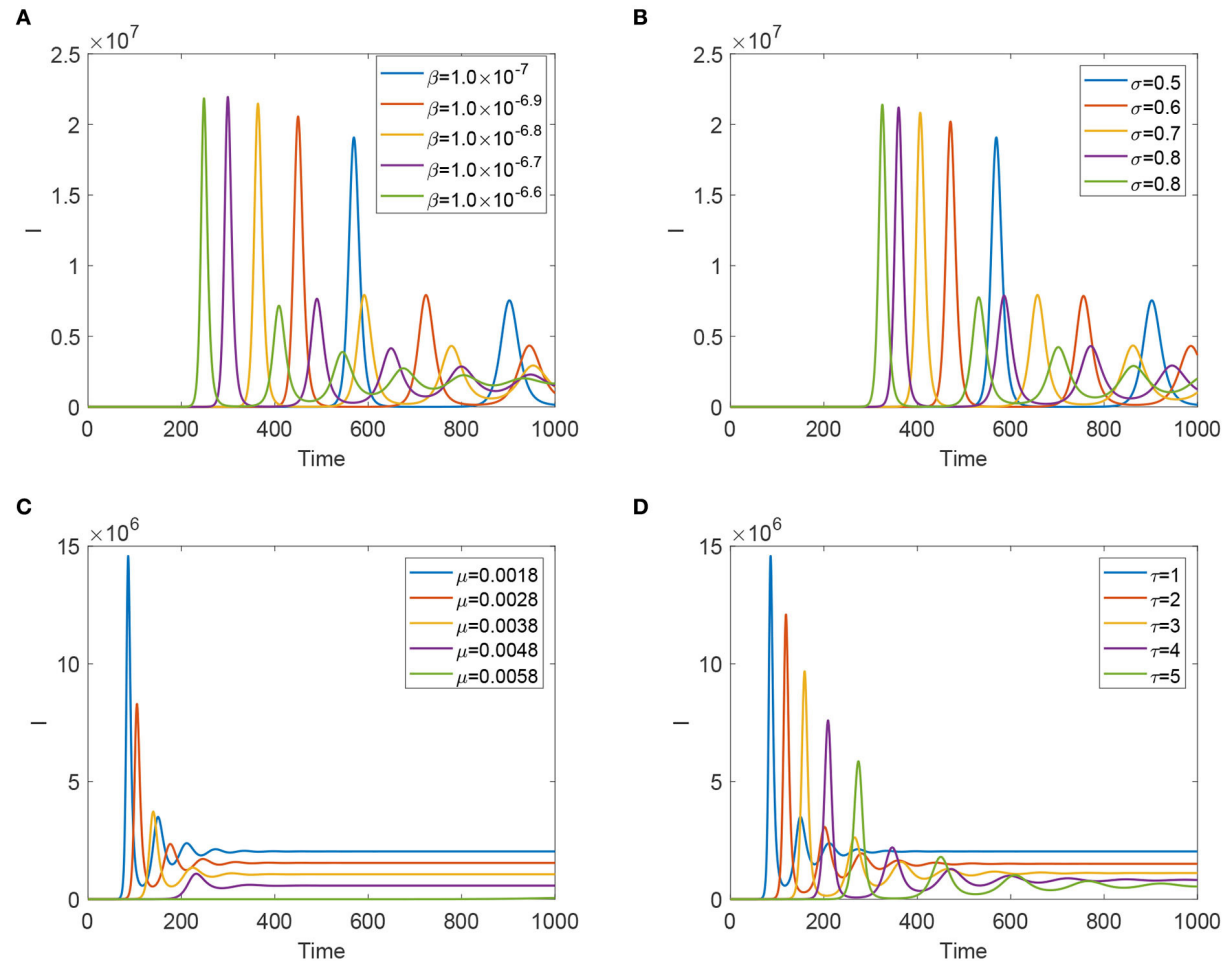
Evolution of infected animals and FMD virus with some significant parameters. **(A)** With different β. **(B)** With different σ. **(C)** With different μ. **(D)** With different τ.

To evaluate each effective control measure, we verify parameters β, μ, σ, and τ to detect the sensitivity analysis of the dynamics of system (Equation 4). From Figures 4A,B, we find that reducing the transmission risk from animal to animal and improving the efficacy of the vaccination can delay the fist peak arrival time and reduce the sizes of peaks, but such two prevention measures enhance the frequencies of temporal oscillations. Figures 4C,D expound that improving the slaughter rate and lengthening the latent period can reduce the size of the final prevalence, delay fist peak arrival time, and decrease the size of each peak. However, lengthening the latent period enhances the frequency of the temporal oscillations; increasing the slaughter rate has a side effect on the frequency of oscillation patterns.

## 4. Conclusion and discussion

This paper proposes a nonlocal, diffusive foot-and-mouth disease model that couples the animal to animal and FMDV-to-animal transmission modes. We derived the basic reproduction number using the next generation operator theory, whose characteristic is equivalent to a principal eigenvalue problem. The basic reproduction number $R_{0}$ is a threshold value determining the outbreak of FMD infection. If $R_{0}<1,$ the disease ends; otherwise, it persists.

**Figure 5:**
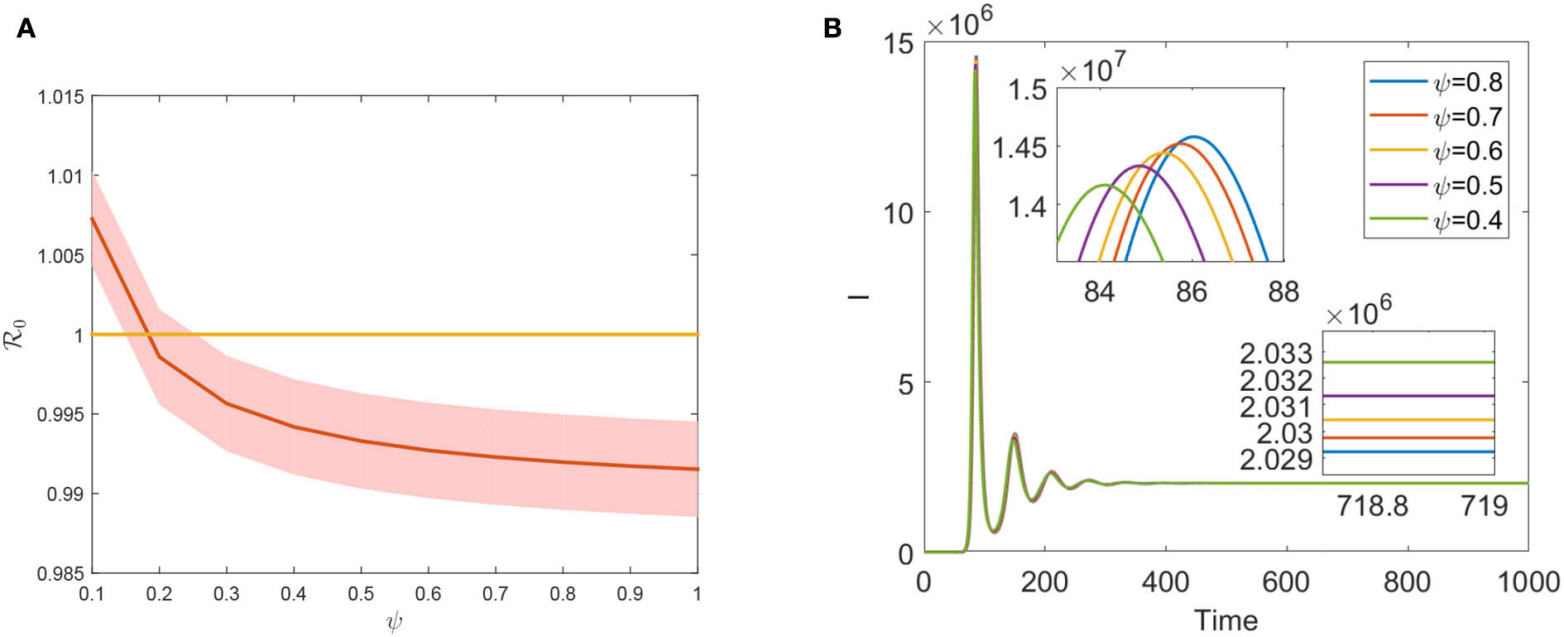
**(A)** The plot of $R_{0}$ *via* ψ. **(B)** Evolution of total infected cattle varies with different ψ.

Vaccination is one of the most important preventive measures for curbing FMD prevalence. However, the evaluation of the FMD vaccination's effectiveness plays a significant role in preventing disease transmission since the vaccine does not provide full immunity against FMD. Theorem 0.0.3 states that $R_{0}<1$ is a necessary condition for eradicating FMD in a region. $R_{0}$ is a declining function concerning ψ, as seen in Figure 5. Hence, ψ effectively decreases the size of $R_{0}$ by increasing vaccination coverage. Moreover, increasing vaccination coverage ψ can delay the first peak arrival period and lower the final prevalence (see Figure 5B). Compared to Figures 4B, 5B, we found that increasing the efficacy of the FMD vaccine has a greater impact on preventing infection than increasing vaccine coverage. The development of more potent vaccines will offer the best defense against FMDV invasion. The first one has a notable accomplishment for reducing the value of $R_{0}$, which suggests that slaughtering the animals and purifying the environment play an effect in the face of an outbreak of an emerging FMD. This contrasts the effects of improving the slaughter rate μ and the vaccination rate ψ. However, such measures will inevitably result in significant economic losses. Long-term, increasing vaccination coverage rates may have a greater economic impact on preventing FMD infection.

**Figure 6:**
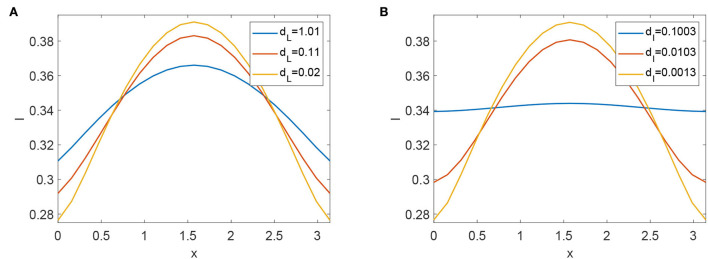
**(A)** Profiles of infected animals with different diffusive coefficients *d*_*L*_. **(B)** Profiles of infected animals with different diffusive coefficients *d*_*I*_.

The reviews of the expression of $R_{0}$ have no relation with any diffusive coefficient. As we know, stochastic movement's speed does have an impact on how FMD transmission scenarios develop. We conducted computational experiments to alter the values of *d*_*L*_ and *d*_*I*_ to understand how diffusive coefficients affect the dynamics of FMD. The scenarios of infected animals eventually flatten (see Figures 6A,B). Increasing the diffusive rate of infected animals is advantageous for reducing the prevalence of FMD.

The carriers of FMDV is defined by confirmed ones if the virus or viral genomes are isolated from the esophageal-pharyngeal fluid more than 28 days after infection. Several experimental evidence shows that carriers may be the main reason of the occasional cause of outbreaks (35, 36). Although the role of carriers in the occurrence of new outbreaks is still a matter of debate (37), it is useful to study the risk of carriers on the persistence of FMDV from a cost-benefit perspective (24) and quantify the risk of infection from carriers to susceptible cattle. We will leave these work in future.

## Data availability statement

The original contributions presented in the study are included in the article/Supplementary material, further inquiries can be directed to the corresponding author.

## Author contributions

JY wrote the manuscript. XW designed the numerical algorithms. KL reviewed the manuscript. All authors contributed to the article and approved the submitted version.

## Funding

This work was partially supported by Humanities and Social Foundation of Ministry of Education of China (22YJAZH129), the National Natural Science Foundation of China (Nos. 12001339 and 12271143), the Shanxi Province Science foundation (20210302123454), and the Shanxi Province Science Foundation for Youths (No. 201901D211413).

## Conflict of interest

The authors declare that the research was conducted in the absence of any commercial or financial relationships that could be construed as a potential conflict of interest.

## Publisher's note

All claims expressed in this article are solely those of the authors and do not necessarily represent those of their affiliated organizations, or those of the publisher, the editors and the reviewers. Any product that may be evaluated in this article, or claim that may be made by its manufacturer, is not guaranteed or endorsed by the publisher.
