# Supplementary material for: Temporal-spatial analysis of a foot-and-mouth disease model with spatial diffusion and vaccination
Source: Front Vet Sci. 2022 Dec 5;9:952382. doi: 10.3389/fvets.2022.952382 (PMC9760958; doi:10.3389/fvets.2022.952382)
Supplement: Supplementary file 1 [file Data_Sheet_1.pdf]

Temporal-spatial analysis of a foot-and-mouth disease model with spatial diffusion and vaccination

In the Appendix, we will give some theoretical results. A diffusive foot-and-mouth disease model with vaccination is taken in form of

$$\begin{cases} \frac{\partial S(t,x)}{\partial t} = d_S \Delta S(t,x) + \Lambda - (\mu + \psi) S(t,x) - S(t,x)\lambda(t,x), x \in \Omega, \\ \frac{\partial V(t,x)}{\partial t} = d_V \Delta V(t,x) + \psi S(t,x) - \mu V(t,x) - \sigma V(t,x)\lambda(t,x), x \in \Omega, \\ \frac{\partial i(t,a,x)}{\partial t} + \frac{\partial i(t,a,x)}{\partial a} = d_i(a)\Delta i(t,a,x) - (\mu + \alpha(a)) i(t,a,x), x \in \Omega, \\ i(t,0,x) = (S(t,x) + \sigma V(t,x)\lambda(t,x), x \in \Omega, \\ \frac{\partial B(t,x)}{\partial t} = d_B \Delta B(t,x) + \int_0^\infty p(a) i(t,a,x) da - c(x) B(t,x), x \in \Omega, \\ \frac{\partial S(t,x)}{\partial \mathbf{n}} = \frac{\partial V(t,x)}{\partial \mathbf{n}} = \frac{\partial i(t,a,x)}{\partial \mathbf{n}} = \frac{\partial B(t,x)}{\partial \mathbf{n}} = 0, x \in \partial\Omega, \end{cases} \tag{S1}$$

S, V and B denotes the densities of susceptible, vaccinated cattle and foot-and-mouth disease virus. i represents the density of infected animals. All the parameters are explained in Table 1 of the main text.

APPENDIX A

In this subappendix, we will drive an equivalent system to reduce the term of age-since-infection. Integrating along the characteristic line $t - a$ =constant, we get

$$i(t,a,x) = \begin{cases} \int_\Omega \Gamma_i(a,x,y) i(t-a,0,y) dy \pi(a), & t \geq a, \\ \int_\Omega \Gamma_i(a,x,y) i_0(a-t,y) dy \frac{\pi(a)}{\pi(a-t)}, & t < a, \end{cases} \tag{S2}$$

where

$$\pi(a) = e^{-\int_0^a (\mu + \alpha(s)) ds}$$

represents the probability of an infected animal survives utile infect age a. The flowchart of model (S1) shows that the infectious state can be divided into two distinct stages: the latent and infected stage. The cattle in the first stage have no transmission ability, while they have the infectious ability in the second stage. Now, we give a clear calculation to derive the equations of such two stage. Deviating E and I with

respect to t, one admits

$$\begin{aligned}\frac{\partial E(t,x)}{\partial t} =& \int_0^\tau \frac{\partial i(t,a,x)}{\partial t}da = -\int_0^\tau \frac{\partial i(t,a,x)}{\partial t}da + \int_0^\tau (d_i(a)\Delta i(t,a,x) - (\mu+\alpha(a))i(t,a,x))da\\ =& i(t,0,x) - i(t,\tau,x) + \int_0^\tau (d_i(a)\Delta i(t,a,x) - (\mu+\alpha(a))i(t,a,x))da,\\ \frac{\partial I(t,x)}{\partial t} =& \int_\tau^\infty \frac{\partial i(t,a,x)}{\partial t}da = -\int_\tau^\infty \frac{\partial i(t,a,x)}{\partial t}da + \int_\tau^\infty (d_i(a)\Delta i(t,a,x) - (\mu+\alpha(a))i(t,a,x))da\\ =& i(t,\tau,x) - i(t,\infty,x) + \int_\tau^\infty (d_i(a)\Delta i(t,a,x) - (\mu+\alpha(a))i(t,a,x))da.\end{aligned}$$

Substituting (S2) into variables E and I leads to

$$\begin{aligned}\frac{\partial E(t,x)}{\partial t} =& \int_0^\tau d_i(a)\Delta i(t,a,x) - \int_0^\tau [\mu+\alpha(a)]da - \int_\Omega \Gamma_i(\tau,x,y)(S(t-\tau,x)+\sigma V(t-\tau,x))\\ &\times \left(\int_0^\infty \beta(a)i(t-\tau,a,y) + \frac{\beta_B B(t-\tau,x)}{1+\alpha B(t-\tau,y)}\right)dy\pi(\tau)\\ &+ (S(t,x)+\sigma V(t,x))\left(\beta(a)i(t,a,x)da + \frac{\beta_B B(t,x)}{1+\alpha B(t,x)}\right), \qquad \text{(S3)}\\ \frac{\partial I(t,x)}{\partial t} =& \int_\tau^\infty d_i(a)\Delta i(t,a,x)da - \int_\tau^\infty [\mu+\alpha(a)]da + \int_\Omega \Gamma_i(\tau,x,y)(S(t-\tau,x)+\sigma V(t-\tau,x))\\ &\times \left(\int_0^\infty \beta(a)i(t-\tau,a,y) + \frac{\beta_B B(t-\tau,x)}{1+\alpha B(t-\tau,y)}\right)dy\pi(\tau).\end{aligned}$$

After some substitutions, we derive a diffusive model with nonlocal infection as follows:

$$\begin{cases}\frac{\partial S(t,x)}{\partial t} = d_S\Delta S(t,x) + \Lambda - (\mu+\psi)S(t,x) - S(t,x)\left(\beta I(t,x) + \frac{\beta_B B(t,x)}{\kappa + v(t,x)}\right), x\in\Omega,\\ \frac{\partial V(t,x)}{\partial t} = d_V\Delta V(t,x) + \psi S(t,x) - \mu V(t,x) - \sigma V(t,x)\left(\beta I(t,x) + \frac{\beta_B V(t,x)}{\kappa + B(t,x)}\right), x\in\Omega,\\ \frac{\partial I(t,x)}{\partial t} = d_I\Delta I(t,x)da - (\mu+\alpha)I(t,x) + \int_\Omega \Gamma_i(\tau,x,y)(S(t-\tau,x)+\sigma V(t-\tau,x))\\ \qquad \times\left(\beta_I I(t-\tau,y) + \frac{\beta_B B(t-\tau,x)}{1+\alpha B(t-\tau,y)}\right)dye^{-(\mu+\alpha)\tau}, x\in\Omega,\\ \frac{\partial B(t,x)}{\partial t} = d_B\Delta B(t,x) + pI(t,x) - cB(t,x), x\in\Omega,\\ \frac{\partial S(t,x)}{\partial \mathbf{n}} = \frac{\partial V(t,x)}{\partial \mathbf{n}} = \frac{\partial i(t,a,x)}{\partial \mathbf{n}} = \frac{\partial B(t,x)}{\partial \mathbf{n}} = 0, x\in\partial\Omega,\end{cases} \qquad \text{(S4)}$$

APPENDIX B

In the appendix, we will show the existence and uniqueness of the positive solution of system (S4). Now, let us introduce a linear operator $A : X \to X$ by

$$A[\phi](\cdot) = (d_S\Delta\phi_1(\cdot) - (\mu + \psi)\phi_1(\cdot), d_V\Delta\phi_2(\cdot) - \mu\phi_2(\cdot), d_I\Delta\phi_3(\cdot) - (\mu + \alpha)\phi_3(\cdot), d_B\Delta\phi_4(\cdot) - c\phi_4(\cdot))^T$$

Besides, define a nonlinear operator $F : C_\tau \to X$ by

$$F[\phi](\cdot) = \begin{pmatrix} \Lambda - \beta\phi_1(0,\cdot)\phi_2(0,\cdot) - \frac{\beta_B\phi_1(0,\cdot)\phi_4(0,\cdot)}{\kappa+\phi_4(0,\cdot)} \\ \psi\phi_1(0,\cdot) - \sigma\phi_4(0,\cdot)\left(\beta\phi_2(0,\cdot) - \frac{\beta_B\phi_4(0,\cdot)}{\kappa+\phi_4(0,\cdot)}\right) \\ \int_\Omega \Gamma_i(\tau,x,y)(\phi_1(-\tau,y) + \sigma\phi_4(-\tau,y))\left(\beta\phi_2(-\tau,y) - \frac{\beta_B\phi_4(-\tau,y)}{\kappa+\phi_4(-\tau,y)}\right) dy e^{-(\mu+\alpha)\tau} \\ p\phi_3(0,\cdot) \end{pmatrix}$$

for any $\phi = (\phi_1, \phi_2, \phi_3, \phi_4) \in C_\tau$. Setting $u = (S, V, I, B)$, then system (S4) can be rewritten as an abstract Cauchy problem

$$\frac{du(t)}{dt} = Au(t) + F(u_t), \quad u(0) = \phi \in C_\tau. \tag{S5}$$

LEMMA 0.1. *For every $\phi = (\phi_1, \phi_2, \phi_3, \phi_4) \in C_\tau^+$, there exists a positive constant T_{max} such that system* (S5) *has unique mild solution $u(t) = u(t, \phi) \in X^+$ defined on $t \in [0, T_{max})$. Moreover, $u(t, x) = u(t, \phi)$ is a classical solution of* (S5) *for all $t > \tau$.*

PROOF. First, noting that the linear operator generates a compact and positive C_0 semigroup $T(t)$ for all $t \geq 0$. From the property of F, it is easy to show that F is Lipschitz continuous. Therefore, it follows from Proposition 4.16 in Webb (1985) that system (S5) has a unique mild solution. Define

$$\Psi(\tau, y) = \beta\phi_2(-\tau, y) + \frac{\beta_B\phi_4(-\tau, y)}{\kappa + \phi_4(-\tau, y)}.$$

Then for any $\phi \in C_\tau^+$ and $h \geq 0$ small enough, one admits

$$\phi(0,\cdot) + hF[\phi](\cdot) = \begin{pmatrix} \phi_1(0,\cdot) + h(\Lambda - \beta\phi_1(0,\cdot)\phi_2(0,\cdot) - \frac{\beta_B\phi_1(0,\cdot)\phi_4(0,\cdot)}{\kappa+\phi_4(0,\cdot)}) \\ \phi_2(0,\cdot) + h(\psi\phi_1(0,\cdot) - \sigma\phi_4(0,\cdot)(\beta\phi_2(0,\cdot) + \frac{\beta_B\phi_4(0,\cdot)}{\kappa+\phi_4(0,\cdot)})) \\ \phi_3(0,\cdot) + h\int_\Omega \Gamma_i(\tau,x,y)(\phi_1(-\tau,y) + \sigma\phi_4(-\tau,y))\Psi(\tau,y)dy e^{-(\mu+\alpha)\tau} \\ \phi_4(0,\cdot) + h(p\phi_2(0,\cdot)) \end{pmatrix}$$

$$\geq \begin{pmatrix} \phi_1(0,\cdot)(1 - h\beta\phi_2(0,\cdot) - h\frac{\beta_B\phi_4(0,\cdot)}{\kappa+\phi_4(0,\cdot)}) \\ \phi_2(0,\cdot)(1 - h\sigma(\beta\phi_2(0,\cdot) - h\sigma\frac{\beta_B\phi_4(0,\cdot)}{\kappa+\phi_4(0,\cdot)})) \\ \phi_3(0,\cdot) \\ \phi_4(0,\cdot) \end{pmatrix},$$

which means $\phi(0, x) + hF[\phi](\cdot) \in C_\tau^+$. Consequently, Theorem 1 in Martin and Smith (1990) ensures that system (S5) has a nonnegative and classical solution.

THEOREM 0.2. *System* (S5) *has a unique solution $u(t, \cdot, \phi) \in C_\tau^+$ defined on $[0, \infty)$.*

PROOF. Noting that for all $(t, x) \in [0, T_{max}) \times \bar{\Omega}$, we have that

$$\frac{\partial S(t,x)}{\partial t} \leq d_S \Delta S(t,x) + \Lambda - \mu S(t,x), x \in \Omega, \tag{S6}$$

$$\frac{\partial S(t,x)}{\partial \mathbf{n}} = 0, x \in \partial\Omega \tag{S7}$$

With the help of the standard parabolic comparison principle, we have known that there exists two positive constants M_S and T_S such that

$$S(t, x, \phi) \leq M_S, \forall (t, x) \in [T_S, +\infty) \times \bar{\Omega}, \phi \in C_\tau^+.$$

From the second equation of (S4), it follows that there exists two positive constants M_V and T_V such that

$$V(t, x, \phi) \leq M_V, \forall (t, x) \in [T_V, +\infty) \times \bar{\Omega}.$$

Define

$$\bar{f}(t) = \int_\Omega f(t, y) dy, f = S, V, I, B.$$

Therefore, for all $t > \tau$,

$$\frac{d(\bar{S}(t-\tau) + \bar{V}(t-\tau) + \bar{I}(t))}{dt} \leq \Lambda|\Omega| - \mu(\bar{S}(t-\tau) + \bar{V}(t-\tau) + \bar{I}(t)) \tag{S8}$$

which suggests that there exists two positive values $\hat{T}$ and $\hat{M}$ such that for all $t \geq \tilde{T} := \{\tau, \hat{T}\}$,

$$\bar{I}(t) \leq \bar{S}(t-\tau) + \bar{V}(t-\tau) + \bar{I}(t) \leq \hat{M}.$$

Employing the last equation of system (S4), one reaches that for $t > \tilde{T}$

$$\frac{d\bar{B}}{dt} \leq p\bar{I} - c\bar{B} \leq p\hat{M} - c\bar{B}, \tag{S9}$$

which indicates that there exists two constants $M_B > 0$ and T_B such that for all $t > T_B$

$$\bar{B}(t) \leq M_B.$$

Similarly, the second equation ensures that there exists two values T_V and M_V such that for all $t > T_B$ $\bar{V}(t) \leq M_T$.

Recalling equation I of system (S4), we have that

$$\frac{\partial I(t,x)}{\partial t} = d_I \Delta I(t,x) da - (\mu + \alpha) I(t,x) + \tilde{M}(\bar{I}(t-\tau) + \bar{B}(t-\tau)), \tag{S10}$$

We have from the boundedness of $\bar{I}$ and $\bar{B}$ that $I(t, x)$ is uniformly bounded. Returning to the last equation of system (S4), we readily have known that B is uniformly bounded. This finished the proof.

Let us introduce two functional spaces $Y = C(\bar{\Omega}, \mathbb{R})$ and $X = Y^4$ with norm

$$\|\psi\|_Y = \max_{x\in\bar{\Omega}} |\psi|, \quad \|\phi\|_X = \sqrt{\|\phi_1\|_Y^2 + \|\phi_2\|_Y^2 + \|\phi_3\|_Y^2 + \|\phi_4\|_Y^2}.$$

They have positive cones $Y_+ = \{\psi \in Y|\psi \geq 0\}$ and $X_+ = Y_+^4$. Moreover, define $C_\tau := C([-\tau, 0], X)$ with norm $\|\phi\| = \max_{\theta\in[-\tau,0]} \|\phi(\theta)\|_X$ and positive cone $C_\tau^+ = C([-\tau, 0], X_+)$. The appendix gives the positivity and uniqueness of the solution of system (S4).

LEMMA 0.3. *The following system*

$$\begin{aligned}
&\frac{\partial S(t,x)}{\partial t} = d_S \Delta S(t,x) + \Lambda - (\mu+\psi)S(t,x), x \in \Omega, t \geq T_B,\\
&\frac{\partial V(t,x)}{\partial t} = d_V \Delta V(t,x) + \bar{S}(t,x) - \mu V(t,x),\\
&\frac{\partial S(t,x)}{\partial \mathbf{n}} = \frac{\partial V(t,x)}{\partial \mathbf{n}} = 0, x \in \partial\Omega.
\end{aligned} \tag{S11}$$

admits a positive equilibrium $\bar{E}^0 = (S^0, V^0) = (\frac{\Lambda}{\mu+\psi}, \frac{p\Lambda}{\mu(\mu+\psi)})$ *which is globally asymptotically stable.*

PROOF. Define a Lyapunov functional as follows

$$W[S,V] = \int_\Omega \left[S^0 g\left(\frac{S(t,x)}{S^0}\right) + V^0 g\left(\frac{V(t,x)}{V^0}\right)\right] dx,$$

where $g(x) = x - 1 - \ln x$ with $x > 0$. Deviating W along the trajectory of (S11), we have that

$$\begin{aligned}
\frac{dW(t)}{dt} =& - d_S \int_\Omega \frac{|\nabla S|^2}{S^2} dx - d_V \int_\Omega \frac{|\nabla V|^2}{V^2} dx - \mu S^0 \int_\Omega \left[g\left(\frac{S(t,x)}{S^0}\right) + g\left(\frac{V(t,x)}{V^0}\right)\right] dx\\
& - \psi S^0 \int_\Omega \left[g\left(\frac{V(t,x)}{V^0}\right) + g\left(\frac{S(t,x)V^0}{S^0 V(t,x)}\right)\right] dx - \psi S^0 \int_\Omega g\left(\frac{S^0}{S(t,x)}\right) dx.
\end{aligned}$$

Obviously, $dW/dt \leq 0$ and the equality holds if and only if $S(t,\cdot) = S^0$ and $V(t,\cdot) = V^0$. From LaSalle invariant principle, it follows that $\bar{E}^0$ is globally asymptotically stable.

The linearizing system of (S4) around the disease free equilibrium E_0 is taken in form of

$$\begin{cases}
\frac{\partial I(t,x)}{\partial t} = d_i \Delta I(t,x) - (\mu+\alpha)I(t,x) + (S^0 + \sigma V^0) \int_\Omega \Gamma_i(\tau, x, y)\\
\qquad \times \left(\beta_I I(t-\tau, y) + \frac{\beta_B B(t-\tau, y)}{\kappa}\right) dy e^{-(\mu+\alpha)\tau}, x \in \Omega,\\
\frac{\partial B(t,x)}{\partial t} = d_B \Delta B(t,x) + pI(t,x) - cB(t,x), x \in \Omega,\\
\frac{\partial I(t,x)}{\partial \mathbf{n}} = \frac{\partial B(t,x)}{\partial \mathbf{n}} = 0, x \in \partial\Omega.
\end{cases} \tag{S12}$$

If $\lambda \in \mathbb{C}$ be an eigenvalue of problem (S12) with associated eigenvector function ϕ_3, ϕ_4, then they satisfy that

$$\begin{cases} \lambda\phi_3(x) = d_i\Delta\phi_3(x) - (\mu+\alpha)\phi_3(x) + (S^0+\sigma V^0)\displaystyle\int_\Omega \Gamma_i(\tau,x,y) \\ \qquad\times\left(\beta_I\phi_3(-\tau,y) + \beta_B\phi_4(-\tau,y)\right)dye^{-(\lambda+\mu+\alpha)\tau}, \\ \lambda\phi_4(x) = d_B\Delta\phi_4(t,x) + p\phi_3(x) - c\phi_4(x), \\ \dfrac{\partial\phi_3(x)}{\partial\mathbf{n}} = \dfrac{\partial\phi_4}{\partial\mathbf{n}} = 0, x\in\partial\Omega. \end{cases} \tag{S13}$$

Setting λ is an eigenvalue of the following problem with same eigenfunctions ϕ_3 and ϕ_4, then they satisfy that

$$\begin{cases} \lambda\phi_3(x) = d_i\Delta\phi_3(x) - (\mu+\alpha)\phi_3(x) + (S^0+\sigma V^0)\displaystyle\int_\Omega \Gamma_i(\tau,x,y) \\ \qquad\times\left(\beta_I\phi_3(y) + \beta_B\phi_4(y)\right)dye^{-(\mu+\alpha)\tau}, \\ \lambda\phi_4(x) = d_B\Delta\phi_4(t,x) + p\phi_3(x) - c\phi_4(x), \\ \dfrac{\partial\phi_3(x)}{\partial\mathbf{n}} = \dfrac{\partial\phi_4}{\partial\mathbf{n}} = 0, x\in\partial\Omega. \end{cases} \tag{S14}$$

By the positivity of parameters $\mu, \alpha, \beta, \beta_B$ Λ and ψ, we have from Crein-Rutman Theorem that problem (S14) has a principal eigenvalue $\lambda_0(\tau, S^0, V^0)$ with a positive eigenfunction.

LEMMA 0.4. *Suppose $\bar\lambda_0(\tau, S^0, V^0)$ is the principal eigenvalue of problem* (S13). *Then $\bar\lambda(\tau, S^0, V^0)$ has the same sign as $\lambda_0(\tau, S^0, V^0)$.*

PROOF. Let us define two functional spaces $E_\tau = C([-\tau, 0], Y^2)$ with positive cone $E_\tau^+ = C([-\tau, 0], Y_+^2)$. Define two linear operator $F : E_\tau \to Y^2$ by

$$\begin{aligned} F_1[\phi](x) =&(S^0+\sigma V^0)\int_\Omega \Gamma_i(\tau,x,y)\times\left(\beta_I\phi_3(-\tau,y)+\frac{\beta_B}{\kappa}\phi_4(-\tau,y)\right)dye^{-(\mu+\alpha)\tau}, \\ F_2[\phi](x) =&p\phi_3(0,x), \end{aligned}$$

Besides, let us introduce a linear operator $L_\lambda : Y^2 \to Y^2$ by

$$\begin{aligned} F_{1\lambda}[\phi](x) =&(S^0+\sigma V^0)\int_\Omega \Gamma_i(\tau,x,y)\times\left(\beta_I\phi_3(y)+\frac{\beta_B}{\kappa}\phi_4(y)\right)dye^{-(\lambda+\mu+\alpha)\tau}, \\ F_{2\lambda}[\phi](x) =&p\phi_3(x), \end{aligned}$$

Then, we can define the following relationship

$$e^{\lambda\cdot}[\phi](\theta,x) = e^{\lambda\theta}[\phi](x),\ \theta\in[-\tau,0], x\in\bar\Omega.$$

Obviously, $s(B+F_0) = \lambda_0(\tau, S^0, V^0)$ and $s(B+F) = \bar\lambda_0(\tau, S^0, V^0)$. The result is a straightforward consequence of Kerscher and Nagel Kerscher and Nagel (1984).

LEMMA 0.5. $\mathcal{R}_0 - 1$ *has the same sign as* $\lambda_0(\tau, S^0, V^0)$.

PROOF. Observing that $L = F + B$ is a positive perturbation of B, we conclude that L generates a C_0 semigroup. Hence, L is resolvent positive. From Lemma 2.2 in Thieme (2009) and Theorem 3.5

in Wang and Zhao (2011), it follows that $s(L) = s(F_0 + B)$ has the same sign as $r(F(-B)^{-1}) - 1 = r(G) - 1 = \mathcal{R}_0 - 1$. This completes the proof.

APPENDIX C

This part gives the detailed proof of Theorem 3.2.

PROOF. First, if $\mathcal{R}_0 < 1$, then we have from Lemma 0.5 that $\lambda_0(\tau, S^0, V^0) < 0$ and moreover, Lemma 0.4 ensures $\bar{\lambda}_0(\tau, S^0, V^0) < 0$. By the continuity of the eigenvalue associated with parameters, we conclude that $\bar{\lambda}_0(\tau, S^0 + \epsilon_0, V^0 + \epsilon_0) < 0$. In view of the fist and second equations, we note that there exists two values T and ϵ_0 such that for all $t > T$ and $x \in \bar{\Omega}$

$$S(t,x) \le S^0 + \epsilon_0, \quad V(t,x) \le V^0 + \epsilon_0.$$

Moreover, by the remaining equations of model (S4), we obtain

$$\begin{cases} \dfrac{\partial I(t,x)}{\partial t} \le d_I \Delta I(t,x) - (\mu+\alpha) I(t,x) + (S^0 + \sigma V^0 + (1+\sigma)\epsilon_0) \displaystyle\int_\Omega \Gamma_i(\tau,x,y) \\ \qquad \times \left(\beta_I I(t-\tau,y) + \dfrac{\beta_B}{\kappa} B(t-\tau,x)\right) dy e^{-(\mu+\alpha)\tau}, \\ \dfrac{\partial B(t,x)}{\partial t} \le d_B \Delta B(t,x) + pI(t,x) - cB(t,x). \end{cases} \tag{S15}$$

Hence, by the comparison principle, we have known that there exists a positive number $\bar{M}$ such that $(I, V) \le \bar{M} e^{\bar{\lambda}_0(\tau, S^0+\epsilon, V^0+\epsilon_0)t}(\psi_3, \psi_4)$ for all $t \ge T - \tau$ where (ψ_3, ψ_4) is the positive eigenfunction associated with $\bar{\lambda}_0(\tau, S^0 + \epsilon_0, V^0 + \epsilon_0)$. If $\mathcal{R}_0 < 1$, then Lemma 0.5 means $\bar{\lambda}_0(\tau, S^0 + \epsilon_0, V^0 + \epsilon_0) < 0$. Hence, $(I(t, x, \phi), B(t, x, \phi)) \to (0, 0)$ as $t \to \infty$. The first and second equations of model (S4) ensure that

$$\lim_{t\to\infty} S(t,x,\phi) = S^0, \quad \lim_{t\to+\infty} V(t,x,\phi) = V^0.$$

In order to establish the second part, let us define

$$W_0 = \{\phi \in C_\tau^+ | \phi_3(0,x) \not\equiv 0 \text{ and } \phi_4(0,x) \not\equiv 0\}$$

and

$$\partial W = C_\tau^+ \setminus W_0 = \{\phi \in C_\tau^+ | \phi_3(0,x) \not\equiv 0 \text{ or } \phi_4(0,x) \not\equiv 0\}.$$

Denote $\Phi(t) : C_\tau^+ \to C_\tau^+$ as the semiflow of (S4), i.e, $\Phi([\phi](\theta, x))(t) = u(t + \theta, x, \phi)$. Set

$$M_\partial = \{\phi \in \partial W | \Phi[\phi](t) \in \partial W, \forall t \ge 0\}.$$

First, we claim that for all $\phi \in M_\partial$, $\omega(\phi) = \{E_0\}$, where $\omega(\phi)$ is the omega limit set of the orbit of Φ through $\phi \in C_\tau^+$.

If $\phi \in M_\partial$, we have that $I(t, \cdot, \phi) \equiv 0$ or $V(t, \cdot, \phi) \equiv 0$. If $I(t, \cdot, \phi) \equiv 0$ for all $t \ge 0$, the last equation of model (S4) gives that $V(t, \cdot, \phi) = 0$ and moreover,

$$\omega(\phi) = \{E_0\}.$$

Similarly, for the case of $V(t,\cdot,\phi)\equiv 0$, the claim readily holds.

If $\mathcal{R}_0 > 1$, Lemma 0.5 suggests that $\bar{\lambda}_0(\tau, S^0, V^0) > 0$. From the continuity of $\bar{\lambda}_0(\tau, S^0, V^0)$, there exists a positive value $\epsilon_0 > 0$ such that $\bar{\lambda}_0(\tau, S^0 - \epsilon_0, V^0 - \epsilon_0) > 0$.

Now, we are in a position to show E_0 is a uniform weak repeller for W_0, i.e,

$$\limsup_{t\to\infty} \|\Phi[\phi] - E_0\| \geq \epsilon_0,\ \forall \phi \in W_0.$$

By way of contradiction, we suppose that there exists $\phi_0 \in W_0$ such that

$$\|\Phi[\phi_0](t) - E_0\| < \epsilon_0.$$

Hence, there exists a positive number t_0 such that for all $(t,x) \in (t_0,+\infty)\times\bar{\Omega}$, $S(t,x) > S^0 - \epsilon$ and $V^0(t,x) > V^0 - \epsilon_0$. Thus I and B satisfy

$$\begin{cases} \dfrac{\partial I(t,x)}{\partial t} \geq d_I \Delta I(t,x) - (\mu+\alpha) I(t,x) + (S^0 + \sigma V^0 - (1+\sigma)\epsilon_0) \displaystyle\int_\Omega \Gamma_i(\tau,x,y) \\ \qquad \times \left(\beta_I I(t-\tau,y) + \dfrac{\beta_B}{\kappa} B(t-\tau,x)\right) dy e^{-(\mu+\alpha)\tau}, x\in\Omega, \\ \dfrac{\partial B(t,x)}{\partial t} = d_B \Delta B(t,x) + pI(t,x) - cB(t,x), x \in \Omega, \\ \dfrac{\partial I(t,x)}{\partial \mathbf{n}} = \dfrac{\partial B(t,x)}{\partial \mathbf{n}}, x\in\partial\Omega, t\geq t_1. \end{cases} \tag{S16}$$

Let us introduce a auxiliary system as follows

$$\begin{cases} \dfrac{\partial \bar{I}(t,x)}{\partial t} = d_I \Delta \bar{I}(t,x) - (\mu+\alpha) I(t,x) + (S^0 + \sigma V^0 - (1+\sigma)\epsilon_0) \displaystyle\int_\Omega \Gamma_i(\tau,x,y) \\ \qquad \times \left(\beta_I \bar{I}(t-\tau,y) + \dfrac{\beta_B}{\kappa} \bar{B}(t-\tau,x)\right) dy e^{-(\mu+\alpha)\tau}, x\in\Omega, \\ \dfrac{\partial \bar{B}(t,x)}{\partial t} = d_B \Delta \bar{B}(t,x) + p\bar{I}(t,x) - c\bar{B}(t,x), x \in \Omega, \\ \dfrac{\partial \bar{I}(t,x)}{\partial \mathbf{n}} = \dfrac{\partial \bar{B}(t,x)}{\partial \mathbf{n}}, x\in\partial\Omega, t\geq t_1. \end{cases} \tag{S17}$$

It is not hard to see that system (S17) has a solution with form of $\epsilon e^{\bar{\lambda}_0(\tau,S^0,V^0)}(\psi_3,\psi_4)$, where (ψ_3,ψ_4) is the positive eigenfunction of $\bar{\lambda}_0(\tau, S^0-\epsilon_0, V^0-\epsilon_0)$ and ϵ is a sufficient small number. From the comparison principle, one arrives at

$$(I(t,x), B(t,x)) \geq \epsilon e^{\bar{\lambda}_0(\tau,S^0,V^0)}(\psi_3,\psi_4),\ \forall (t,x)\in(t_0,\infty)\times\bar{\Omega}.$$

This is a contradiction with the boundedness of I and B and hence the claim doesn't hold.

Define a continuous function $f: C_\tau^+ \to \mathbb{R}_+$ by

$$f[\phi] := \min \min_{x\in\bar{\Omega}} \{\phi_3(0,x), \phi_4(0,x)\}$$

From the positivity of the solution of system (S4), it follows that $f^{-1}(\mathbb{R}_+) \subset W_0$ and f is a generalized distance function for the semiflow Φ. From what has been discussed, for any forward orbit of $\Phi(t)(M_\partial) \to E_0$ and it is isolated in C_τ^+ and $W^S(E_0) \cap W_0 = \emptyset$, where $W^S(E_0)$ is the stable manifold of E_0. Theorem 3 Smith and Zhao (2001) indicates that there exists an $\bar{\epsilon} > 0$ such that

$$\min_{\psi \in \omega(\phi)} f[\psi] > \tilde{\epsilon},\ \forall \phi \in W_0.$$

Hence,

$$\lim_{t\to\infty}\inf I(t,\cdot) \geq \tilde{\epsilon},\ \lim_{t\to\infty}\inf B(t,\cdot) \geq \tilde{\epsilon}.$$

From Lemma 3.1, there exists an $\epsilon_0 \leq \tilde{\epsilon}$ such that

$$\lim_{t\to\infty}\inf S(t,\cdot) \geq \tilde{\epsilon},\ \lim_{t\to\infty}\inf V(t,\cdot) \geq \tilde{\epsilon}.$$

The uniform persistence readily holds. Employing Theorem 3.7 and Remark 3.10 in Magal and Zhao (2005), we have conclude that $\Phi : W_0 \to W_0$ has a globally attractor $\mathcal{A}_0$. Theorem 4.7 Smith and Zhao (2001) suggests that Φ has an at least one equilibrium $E^* \in W_0$.

REFERENCES

Kerscher, W. and Nagel, R. (1984). *Asymptotic behavior of one-parameter semigroups of positive operators, in "Positive Semigroups of Operators, and Applications"* (New York: Springer-Verlag)

Magal, P. and Zhao, X. (2005). Global attractors and steady states for uniformly persistent dynamical systems. *SIAM J. Appl. Math.* 37, 251–275

Martin, R. and Smith, H. (1990). Abstract functional-differential equations and reaction-diffusion systems. *Trans. Amer. Math. Soc.* 321, 1–44

Smith, H. and Zhao, X. (2001). Robust persistence for semidynamical systems. *Nonlinear Anal.* 47, 6169–6179

Thieme, H. (2009). Spectral bound and reproduction number for infinite-dimensional population structure and time heterogeneity. *SIAM J. Appl. Math.* 70, 188–211

Wang, W. and Zhao, X. (2011). A nonlocal and time-delayed reaction-diffusion model of dengue transmission. *SIAM J. Appl. Math.* 71, 147–168

Webb, G. (1985). *Theory of Nonlinear Age-Dependent Population Dynamics* (New York: Marcel Dekker Inc.)
